# PvMSP8 as a Novel *Plasmodium vivax* Malaria Sero-Marker for the Peruvian Amazon

**DOI:** 10.3390/pathogens10030282

**Published:** 2021-03-02

**Authors:** Elizabeth Villasis, Katherine Garro, Angel Rosas-Aguirre, Pamela Rodriguez, Jason Rosado, Anthony Gave, Mitchel Guzman-Guzman, Paulo Manrique, Michael White, Niko Speybroeck, Joseph Michael Vinetz, Katherine Torres, Dionicia Gamboa

**Affiliations:** 1 Laboratorio de Malaria, Laboratorios de Investigación y Desarrollo, Facultad de Ciencias y Filosofía, Universidad Peruana Cayetano Heredia, 15102, Lima, Peru; katherine.garro@upch.pe (K.G.); pamela.rodriguez@upch.pe (P.R.); katherine.torres.f@upch.pe (K.T.); 2 Instituto de Medicina Tropical Alexander von Humboldt, Universidad Peruana Cayetano Heredia, Lima 15102, Peru; angel.rosas@uclouvain.be (A.R.-A.); joseph.vinetz@yale.edu (J.M.V.); dionicia.gamboa@upch.pe (D.G.); 3 Research Institute of Health and Society (IRSS). Université Catholique de Louvain, Clos Chapelle-aux-champs 30/B1.30.14 1200 Woluwe-Saint-Lambert, Brussels 1200, Belgium; niko.speybroeck@uclouvain.be; 4 Malaria: Parasites and Hosts Unit, Institut Pasteur, Paris 75015, France; javier.rosado@pasteur.fr (J.R.); michael.white@pasteur.fr (M.W.); 5 Sorbonne Université, Faculté des Sciences et Ingénierie, École Doctorale Pierre Louis - Santé Publique, Campus des Cordeliers, ED 393, F-75005 Paris, France; 6 Laboratorio de Malaria: Parásitos y Vectores, Laboratorios de Investigación y Desarrollo, Facultad de Ciencias y Filosofía, Universidad Peruana Cayetano Heredia, Lima 15102, Peru; anthony.gave.z@upch.pe; 7 Laboratorio ICEMR-Amazonia, Laboratorios de Investigación y Desarrollo, Facultad de Ciencias y Filosofía, Universidad Peruana Cayetano Heredia, Lima 15102, Peru; guzman.mitch@gmail.com; 8 Leishmania and Malaria Research Unit. Instituto de Medicina Tropical Alexander von Humboldt, Universidad Peruana Cayetano Heredia, Lima 15102, Peru; paulonvnv@gmail.com; 9 Section of Infectious Diseases, Department of Internal Medicine, Yale School of Medicine, New Haven, CT 06520, USA; 10 Departamento de Ciencias Celulares y Moleculares, Facultad de Ciencias y Filosofía, Universidad Peruana Cayetano Heredia, Lima 15102, Peru

**Keywords:** PvMSP8, antibodies, *P. vivax*, Luminex, ELISA, malaria

## Abstract

The measurement of recent malaria exposure can support malaria control efforts. This study evaluated serological responses to an in-house *Plasmodium vivax* Merozoite Surface Protein 8 (PvMSP8) expressed in a Baculovirus system as sero-marker of recent exposure to *P. vivax* (Pv) in the Peruvian Amazon. In a first evaluation, IgGs against PvMSP8 and PvMSP10 proteins were measured by Luminex in a cohort of 422 Amazonian individuals with known history of Pv exposure (monthly data of infection status by qPCR and/or microscopy over five months). Both serological responses were able to discriminate between exposed and non-exposed individuals in a good manner, with slightly higher performance of anti-PvMSP10 IgGs (area under the curve AUC = 0.78 [95% CI = 0.72–0.83]) than anti-PvMSP8 IgGs (AUC = 0.72 [95% CI = 0.67–0.78]) (*p* = 0.01). In a second evaluation, the analysis by ELISA of 1251 plasma samples, collected during a population-based cross-sectional survey, confirmed the good performance of anti-PvMSP8 IgGs for discriminating between individuals with Pv infection at the time of survey and/or with antecedent of Pv in the past month (AUC = 0.79 [95% CI = 0.74–0.83]). Anti-PvMSP8 IgG antibodies can be considered as a good biomarker of recent Pv exposure in low-moderate transmission settings of the Peruvian Amazon.

## 1. Introduction

As observed in different malaria-endemic countries, important reductions in malaria incidence can be achieved with intensive and comprehensive control measures [1]. However, these reductions are not often homogenous in *P. falciparum* and *P. vivax* co-endemic countries, commonly observing a greater and faster impact of control efforts in the *P. falciparum* burden when compared to *P. vivax* [2]. In line with the long-term malaria goals set by the World Health Organization (WHO), the Peruvian Government launched the “Malaria Zero Plan” initiative, aiming to eliminate malaria in the country by 2042 [3]. Since 2017, intensive control measures (i.e., delivery of long-lasting insecticide nets, indoor residual spraying, active case detection (ACD) and treatment of individuals with microscopically confirmed infections) have been deployed in several communities in the Peruvian Amazon, with the goal of reducing malaria incidence by more than 70% in a couple of years (first phase of the plan). Interestingly, the number of reported malaria cases in 2019 decreased by almost 47% in comparison with 2018, observing an increase in the ratio between *P. vivax* and *P. falciparum* cases from 3.6/1 in 2018 to 4.6/1.9 in 2019 [4,5].

*P. vivax* has several biological characteristics that largely explain its resilience to malaria control and elimination efforts. The early development of infectious blood-stage parasites (i.e., mature gametocytes) before clinical symptoms arise, makes *P. vivax* highly transmissible. In addition, asymptomatic and low-density blood-stage infections are very common, especially in low transmission areas, making *P. vivax* diagnosis by light microscopy (LM) or rapid diagnostic tests (RDTs) particularly difficult. Furthermore, the capability of *P. vivax* to develop a latent liver stage (hypnozoites), that can cause relapses following primary infections, also represents an important challenge for diagnostic since this stage cannot be detected by currently diagnostic methods [6].

The measurement of recent malaria exposure using antibodies can support the decision-making to improve or re-orient the strategies to control malaria and eventually, eliminate it [7]. Since antibodies against parasite proteins are able to persist in blood (even after blood parasite clearance by treatment), they can be used as serological exposure markers (SEMs) to identify ongoing and past infections. National Malaria Control Programs (NMCPs) can use SEM to document the absence of malaria transmission in defined areas, stratify the risk of transmission, assess the effect of interventions, inform of an immediate response to malaria control teams in the field, and identify and treat individuals who potentially carry *P. vivax* hypnozoites [7]. Regarding this latter potential application, a panel of eight SEM, able to induce antibodies in individuals with *P. vivax* exposure within the previous nine months, has recently been validated using samples of several endemic areas and proposed to target treatment to people at risk of carrying clinically silent hypnozoites [8]. 

PvMSP1_19_ and PvAMA1 are among the most used *P. vivax* SEMs in epidemiological studies in co-endemic areas for *P. vivax* and *P. falciparum* [9,10,11,12]. They have proven to characterize malaria transmission changes at micro-geographical level in the Peruvian Amazon [13]. The first study conducted in the Peruvian Amazon, to identify more *P. vivax* SEMs, profiled IgG antibody responses against more than 3000 *P. vivax* antigens, through the use of protein arrays on plasma samples of symptomatic individuals, identified *P. vivax* Merozoite Surface Protein-10 (PvMSP10) and *P. vivax* Merozoite Surface Protein-8 (PvMSP8) among the top five most seroreactive proteins in this large scale analysis [14]. Recently, the evaluation of IgG antibody responses against mammalian cell-produced recombinant protein PvMSP10, measured by enzyme-linked immunosorbent assay (ELISA) on plasma samples of cohort participants with a five-month known history of malaria infection (Amazonian Center of Excellence in Malaria Research (AICEMR) cohort), confirmed the potential of this protein as a serosurveillance marker for recent *P. vivax* exposure in the co-endemic Amazonian region [15].

The present study was designed to test the hypothesis that IgG responses to an in-house baculovirus expressed the PvMSP8 recombinant protein would be a good SEM for the identification of individuals with recent exposure to *P. vivax* at the population level in the Peruvian Amazon. After successfully producing a recombinant PvMSP8 protein in our laboratory using the Baculovirus expression system, we evaluated the performance of IgG responses against this protein in discriminating between individuals with recent *P. vivax* infection and those without infection. In a first evaluation using the same samples of the AICEMR cohort study [15], IgG responses against PvMSP8 and PvMSP10 were measured by Luminex and their performance to discriminate *P. vivax* exposure of different times (since last infection) were compared. Then, in a second evaluation, anti-PvMSP8 IgGs responses measured by ELISA were assessed in terms of their ability to identify *P. vivax* recent infections at the population level in the Peruvian Amazon.

## 2. Results

### 2.1. Preliminary Assessment after PvMSP8 Protein Production

**Figure 1:**
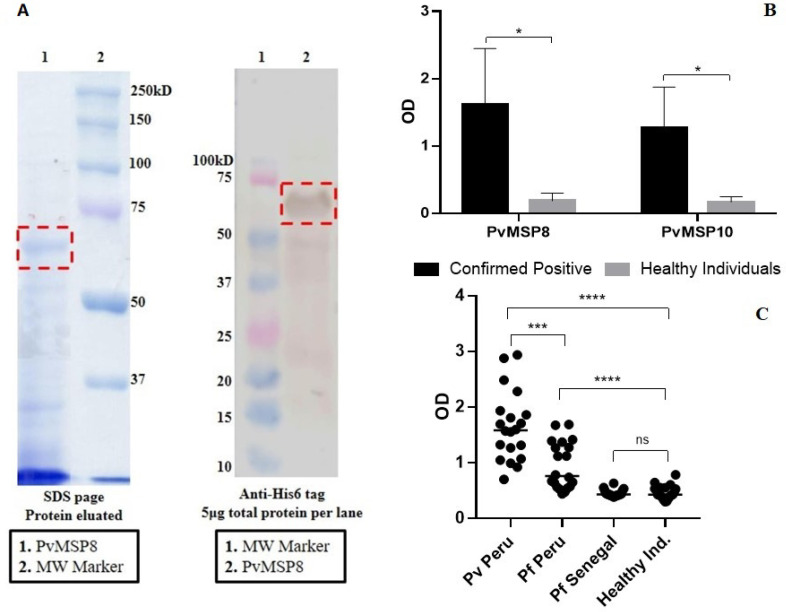
Evaluation of PvMSP8 protein production. (**A**) PvMSP8 recombinant protein production. Recombinant protein was affinity-purified from culture supernatants with nickel affinity chromatography. SDS-PAGE (Coomassie Blue staining) and Western blot (using anti-His6 monoclonal antibody) analysis demonstrated proteins of the expected size (54 kDa). (**B**) Evaluation of IgG responses against PvMSP8 and PvMSP10 recombinant protein by ELISA. Total IgG levels of plasma samples from confirmed *P. vivax* positive individuals in comparison with healthy individuals. (**C**) Cross-reactivity assays for the evaluation of PvMSP8 specific IgG antibodies responses by ELISA. Total IgG levels in plasma samples from *P. falciparum*-infected Senegalese subjects (Pf Senegal), *P. falciparum*-infected individuals from the Peruvian Amazon (Pf Peru), *P. vivax*-infected individuals from the Peruvian Amazon (Pv Peru), and healthy individuals against PvMSP8 were compared. The horizontal line for each group shows the median. The statistical significance of a difference between groups is indicated with upper horizontal branches. * *p* < 0.05, *** *p* < 0.001; **** *p* < 0.0001, ns no significant.

PvMSP8 recombinant protein was produced in the Malaria Laboratory of UPCH using a Baculovirus expression system. Western blot assays, using an anti-His detection antibody, confirmed the presence of a band containing  ~ 54 kDa protein, which corresponded to the molecular weight of PvMSP8 (Figure 1A). The production was 0.428mg/mL of protein in a *Spodoptera frugiperda* (Sf9) cell culture volume of 170 mL. A preliminary ELISA assay confirmed the protein antigenicity of produced PvMSP8, after finding higher Optical Density (OD) values in plasma samples from individuals with confirmed *P. vivax* mono-infection by qPCR (*n* = 40), when compared with plasma samples from healthy individuals with no history of malaria infection and negative qPCR (*n* = 40) (Mann–Whitney test *p* < 0.05) (Figure 1B).

Since *P. falciparum* and *P. vivax* infections coexist in the Peruvian Amazon region, another ELISA assay was performed to assess potential cross-reactivity between recombinant PvMSP8 and *P. falciparum* plasma. Anti-PvMSP8 OD values, obtained from plasma samples, were not different between *P. falciparum*-infected Senegalese subjects (*n* = 20) and healthy individuals (*p* = 0.91). However, this was not the case when comparing samples from *P. falciparum*-infected individuals in the Peruvian Amazon and negative controls (higher OD values in infected ones, *p* < 0.001), or samples from *P. falciparum* (*n* = 20) and *P. vivax*-infected individuals (*n* = 20) both from the Peruvian Amazon (higher OD values in infected *P. vivax* ones, *p* < 0.001) (Figure 1C).

### 2.2. Evaluation Using Samples of Individuals with Known History of P. vivax Exposure (Cohort Study)

**Figure 2:**
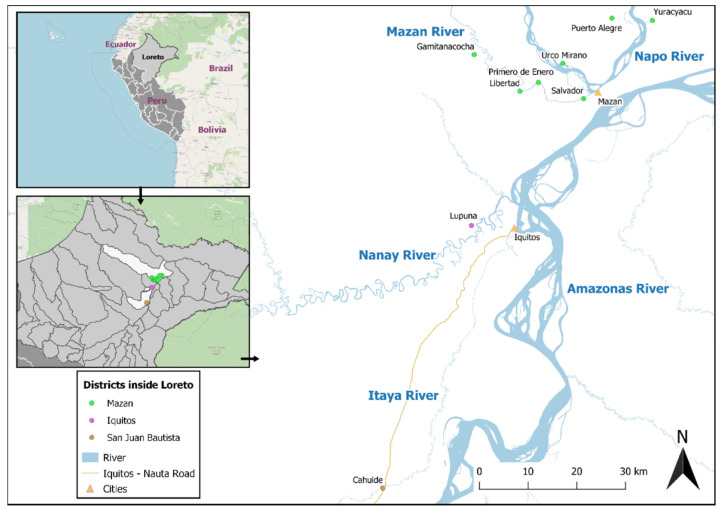
District’s location of communities.

Plasma samples collected at the end of a longitudinal cohort study (January 2013) in two Amazonian communities near Iquitos city (Figure 2) [15] were analyzed by Luminex to measure antigen-specific IgG antibody responses against two recombinant proteins: the Baculovirus-produced PvMSP8 and the mammalian cell–produced PvMSP10. The follow-up of participants (*n* = 422) in this cohort had a median duration of 117 days (range = 95–175 days). During this cohort period, about one third of participants (*n* = 138, 32.7%) had *P. vivax* infection confirmed qPCR and/or microscopy; therefore, these participants were considered as recently exposed *P. vivax* individuals, while the remaining participants were considered as non-exposed individuals. Baseline characteristics of study participants are presented in Table S1.

The median time between the last *P. vivax* infection and end of follow-up was 48.5 days (range = 0–167 days). At the time of plasma collection, cohort data showed that 11 individuals had *P. vivax* parasite confirmation in the previous six days, 28 between seven and 30 days before, 56 between 31 and 60 days before, and 43 individuals between 61 and 169 days before. The majority of individuals with *P. vivax* episodes (54.4%, 75/138) did not have any malaria-compatible symptom when they were diagnosed.

**Figure 3:**
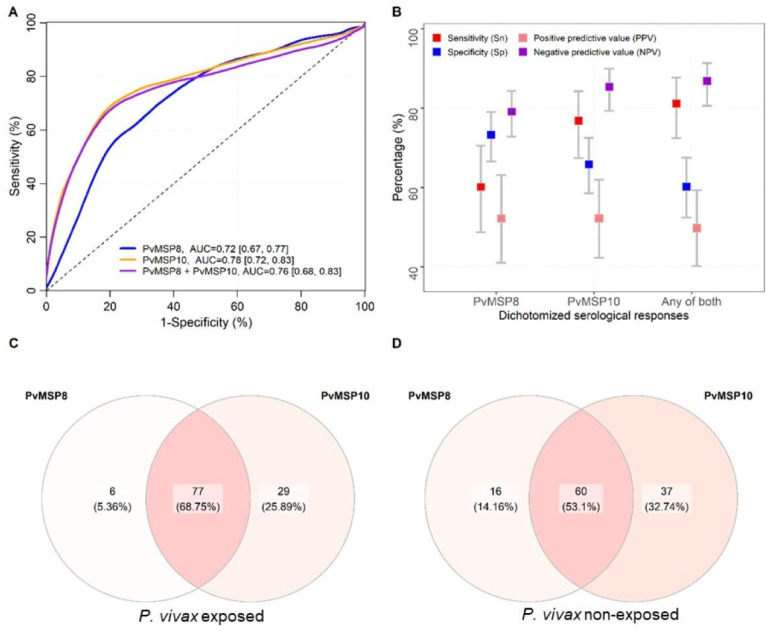
Performance of serological responses against PvMSP8 and PvMSP10 for detecting *P. vivax* exposure. (**A**) Cross-validated Receiver Operating Characteristic (ROC) curves and Areas Under the Curve (AUCs) of serological responses against PvMSP8, PvMSP10, and the combination of both antigens for detecting *P. vivax* exposure. *P. vivax* exposure were defined by a *P. vivax*-positive result by LM and/or qPCR at any time during the five-month follow-up before plasma collection. Serological responses were measured by Luminex in 422 samples. (**B**) Performances indicators of dichotomized serological responses against PvMSP8 and PvMSP10 for detecting *P. vivax* exposure. Cutoff for seropositivity was the mean plus three standard deviations of negative controls. Performance indicators were also calculated for dichotomized responses to any of both antigens. (**C**) Venn diagram showing the number and percentage of seropositive individuals to one or both antigens among *P. vivax*-exposed individuals with seropositivity to any of both antigens. (**D**) Venn diagram showing the number and percentage of seropositive individuals to one or both antigens among non-exposed *P. vivax* individuals with seropositivity to any of both antigens.

The cross-validated receiver operating characteristic (ROC) analysis showed a good performance of serological responses against PvMSP8 for detecting *P. vivax* recent exposure (area under the curve (AUC = 0.72 [95% CI = 0.67–0.77]), which was slightly lower than the performance of anti-PvMSP10 (AUC = 0.78 [95% CI = 0.72–0.83]; *p* = 0.01). The combination of both proteins in the analysis did not improve the discrimination between Pv exposed and non-exposed individuals (AUC = 0.76 [95% CI = 0.67–0.86]) (Figure 3A) [16]. Seropositivity to PvMSP8 (using the conventional cutoff of the mean plus three standard deviation of negative controls) identified individuals who acquired *P. vivax* infections with a moderate sensitivity (Sn) of 60.1% (95% CI = 48.8–70.6), specificity (Sp) of 73.2% (95% CI = 66.6–79.0), positive predictive value (PPV) of 52.2% (95% CI = 41.0–63.2), and negative predictive value (NPV) of 79.1% (95% CI = 72.8–84.3). Comparatively, seropositivity to PvMSP10 showed higher Sn 76.8% (95% CI = 67.4–84.2) and NPV 85.4% (95% CI = 79.3–90.0), but a lower Sp 65.8% (95% CI = 58.5–72.5), PPV was 52.2% (95% CI = 42.3–61.9). Seropositivity to any of both antigens showed a higher Sn 81.2% (95% CI = 72.4–87.7) but a lower Sp 60.2% (95% CI = 52.4–67.5), PPV was 49.8% (95% CI = 40.2–59.3) and NPV 86.8% (95% CI = 80.6–91.3) (Figure 3B). Interestingly, among all samples of individuals with confirmed *P. vivax* exposure and seropositivity to any of both antigens, the majority of them were seropositive to both antigens (Figure 3C). The same occurred when seropositive samples of individuals that did not have *P. vivax* infections were analyzed (Figure 3D)

**Figure 4:**
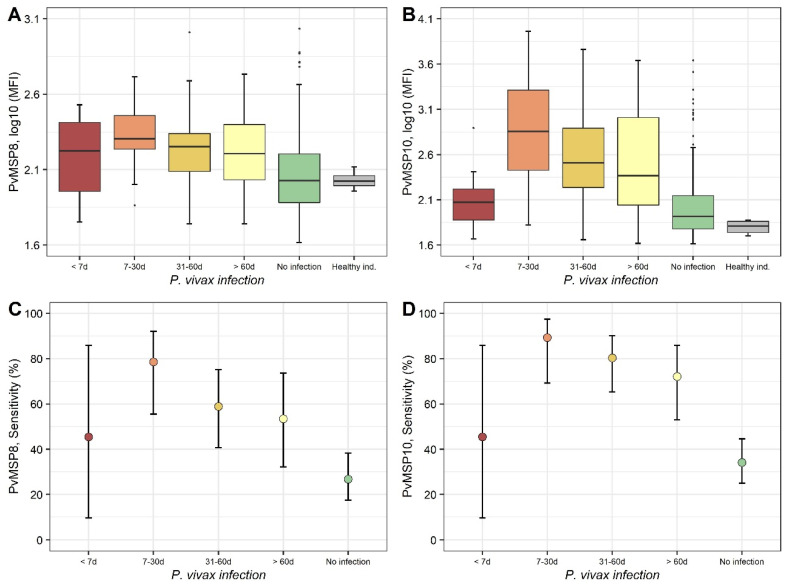
Time-dependent decay kinetics of serological responses against PvMSP8 and PvMSP10 since last *P. vivax* infection. (**A**,**B**) Box plots (25th–75th percentiles) of IgG values (log_10_MFI) obtained by Luminex for PvMSP8 and PvMSP10. Horizontal bars inside the boxes represent the median of log_10_MFI values in each group. (**C**,**D**) Dot plots of the sensitivity of dichotomized serological results for PvMSP8 and PvMSP10 in detecting malaria exposure, showing mean and 95% confidence limits in each group estimated by the Wilson score method.

Individuals with infections between seven and 30 days before plasma sample collection had the highest IgG values (log_10_ of median fluorescence intensity (MFI) values) against PvMSP8 (median = 2.31, and IQR = 0.22) and then this response decreased with the time since last infection, being 31–60 days (median = 2.25, and IQR = 0.25) and more than 60 days (median = 2.21, and IQR = 0.37) (Kruskal–Wallis test, *p* < 0.001) (Figure 4A). The pairwise comparison found that individuals with detected infections (except for individuals with infections less than seven days due to low sample numbers) had higher IgG levels than individuals without infections (post hoc Dunn’s test, *p* < 0.05) and that the median of this latter group (2.02 and IQR = 0.32) did not differ from healthy individuals (2.02 IQR = 0.07) (*p* > 0.05). IgG log_10_ MFI values against PvMSP10 were also higher in individuals with infections between seven and 30 days before plasma sample collection (median = 2.86, and IQR = 0.88) and also decreased with the time since last infection 31–60 days (median = 2.44, and IQR = 0.69) and more than 60 days (median = 2.33, and IQR = 1.05) (Kruskal–Wallis test, *p* < 0.001) (Figure 4B). Similar to PvMSP8, pairwise comparison found that with exception of infections less than seven days, all other infections had higher anti-PvMSP10 IgG levels (median 2.44 and IQR = 0.88) than individuals without infections (median 1.88 and IQR = 0.36) (post hoc Dunn’s test, *p* < 0.001). IgG levels against PvMSP10 in individuals with no-infection (median 1.88 and IQR = 0.36) did not differ from those in healthy individuals (1.81 IQR = 0.12) (*p* > 0.05).

The sensitivity (Sn) of dichotomized serological results for detecting *P. vivax* exposure using PvMSP8 was 45% (95% CI = 10–86) on plasma samples from individuals with *P. vivax* infections of less than seven days before plasma collection and increased to 79% (95% CI = 56–92) with infections between seven and 30 days, but then Sn decreased after 31–60 days (59%, 95% CI = 41–75) and more than 60 days (53%, 95% CI = 32–74) (Figure 4C). A similar time-based pattern was observed for the Sn of seropositivity to PvMSP10 with the time since last infection, with Sn of 45% (95% CI = 10–86) in infections of less than seven days before plasma collection, an increase to 89% (95% CI = 69–97) in infections between seven and 30 days, and a decrease with older infections of 31–60 days (80%, CI = 65–90) and of more than 60 days (72%, 95% CI = 53–86) (Figure 4D).

### 2.3. Evaluation Using Samples Collected in a Population-Based Cross-Sectional Survey

Plasma samples collected during a population-based cross-sectional survey in seven communities of Mazan district (October 2018) were analyzed by ELISA to measure antigen-specific IgG antibody responses against the Baculovirus-produced PvMSP8 protein. The baseline characteristics of the 1251 surveyed participants are shown in Table S2. Females slightly outnumbered males (ratio female/male = 1.06); half (50.8%) of the population was less than 15 years old.

Only 81 (6.5%) participants had *P. vivax* malaria confirmed by microscopy and/or qPCR at the time of plasma sample collection, thus composing a subgroup of exposed individuals to *P. vivax* based on only their parasitological diagnosis (first definition of *P. vivax* exposure). Thirty (2.4%) additional participants reported to have a microscopically-confirmed *P. vivax* malaria episode in the past month, which added to the before mentioned subgroup conformed a total of 111 exposed individuals to *P. vivax* based on their parasitological diagnosis at the time of the survey and/or history of malaria in the past month (second definition of *P. vivax* exposure). The composition of exposed and non-exposed groups according to the two definitions used for *P. vivax* exposure is shown in Table S3.

**Figure 5:**
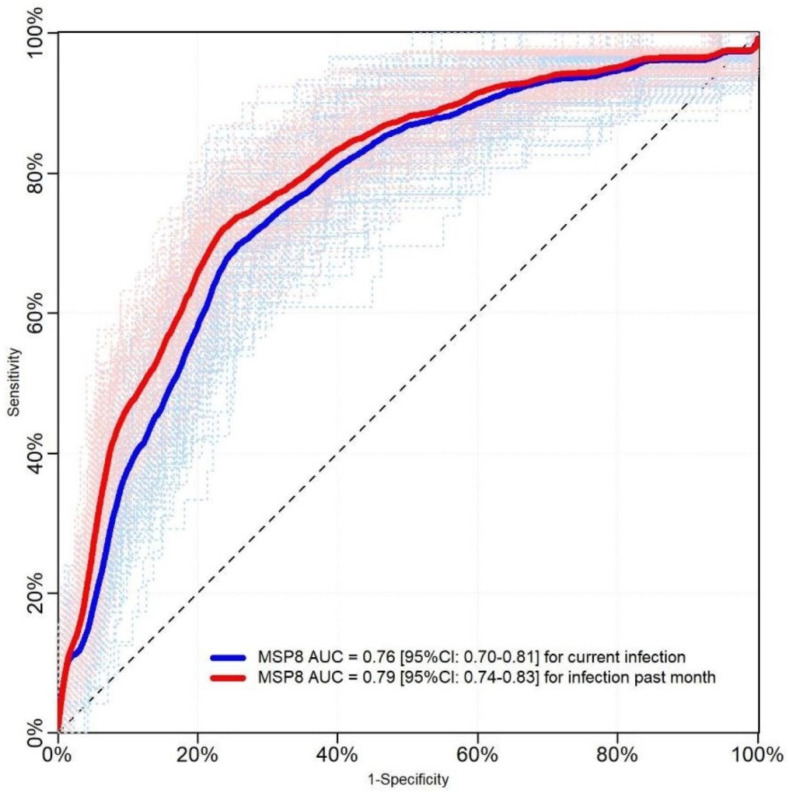
Cross-validated Receiver Operating Characteristic (ROC) curves and Areas Under the Curve (AUCs) of serological responses against PvMSP8 for detecting malaria exposure. Serological responses against PvMSP8 were measured by ELISA in a population of 1251 individuals in riverine communities in Mazan. A first definition of *P. vivax* exposure was a positive *P. vivax* result (by microscopy and/or qPCR) at the time of the sample collection (blue line), while a second definition included individuals with a positive result and/or the antecedent of *P. vivax* malaria in the past month (red line). No significant difference between results was found (*p* > 0.05).

**Figure 6:**
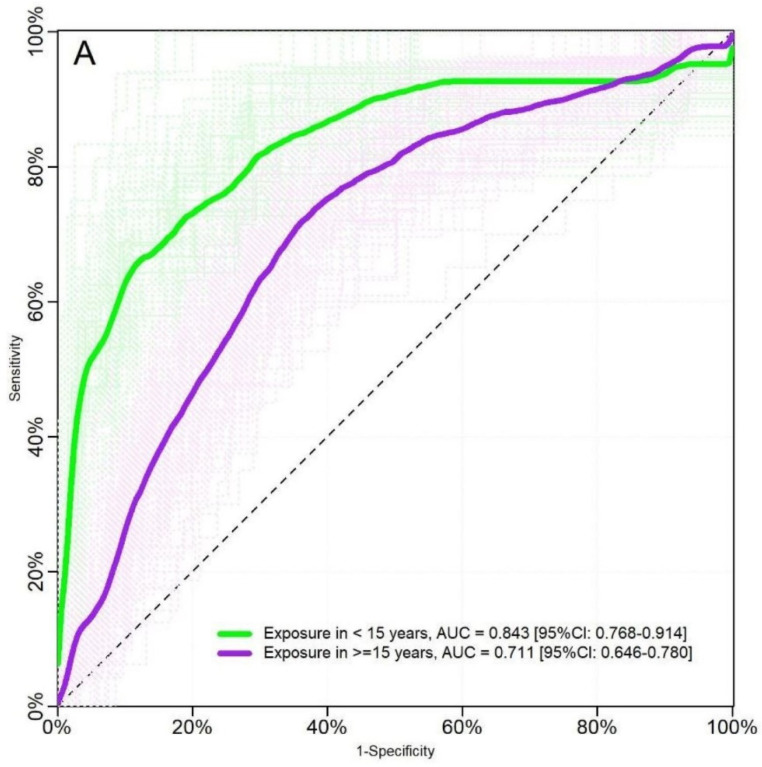
Cross-validated Receiver Operating Characteristic (ROC) curves and Areas Under the Curve (AUCs) of serological responses against PvMSP8 for detecting *P. vivax* malaria exposure among individuals < 15 years old and among those >15 years old. Significant difference between results were found (*p* = 0.01). Exposure was defined as a confirmed *P. vivax* diagnosis by qPCR and/or microscopy and the antecedent of *P. vivax* malaria in the past month (second definition).

PvMSP8 responses for discriminating between exposed and non-exposed *P. vivax* individuals yielded an AUC of 0.76 ([95% CI = 0.70–0.81]) when the first definition of exposure was used, and an AUC of 0.79 ([95% CI = 0.74–0.83]) when the second definition was used (*p* > 0.05) (Figure 5). Moreover, the ROC curve analyses by age groups (keeping the second definition of *P. vivax* exposure) showed that anti-PvMSP8 discriminated better between exposed and non-exposed individuals among participants <15 years (AUC = 0.843 [95% CI = 0.768–0.914]) than in those ≥ 15 years (AUC = 0.711 [95% CI = 0.746–0.780]) (Figure 6).

**Figure 7:**
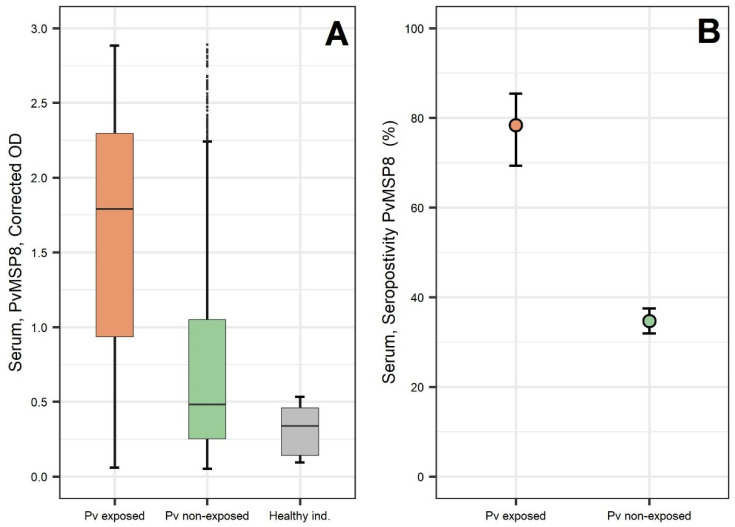
Serological responses against PvMSP8 in exposed and non-exposed individuals from Mazan communities. (**A**) Boxplots (25th–75th percentiles) of corrected optical density values for PvMSP8. Horizontal bars inside the boxes represent the median of Optical Density (OD) values in each group; (**B**) Dot plots of the sensitivity of dichotomized serological results for PvMSP8 in detecting malaria exposure, showing mean and 95% confidence limits in each group estimated by the Wilson score method. Exposure was defined as a confirmed *P. vivax* diagnosis by qPCR and/or microscopy parasitological and the antecedent of *P. vivax* malaria in the past month.

Corrected anti-PvMSP8 ODs were significantly higher in exposed individuals (median = 1.78, and IQR = 1.37) when compared to individuals without exposure (median = 0.48, and IQR = 0.80), and healthy individuals (median = 0.34, and IQR = 0.32) (Kruskal–Wallis test, *p* < 0.001). Similarly, seropositivity to PvMSP8 was significantly higher in the exposed group (77%, 95% CI = 67–84) when compared to the unexposed group (31%, 95% CI = 29–34) (*p* < 0.001) (Figure 7).

Supplementary material Figure S1 shows the variation of the sensitivity (Sn), specificity (Sp), positive likelihood ratio (PLR) and negative likelihood ratio (NLR) of PvMSP8 IgG in discriminating *P. vivax* exposure according to different cut-offs to define seropositivity. Lower cut-offs improve the Sn and decrease the odds of being exposed in an individual with a negative serological result (lower NLR), while higher cut-offs improve the Sp and the odds of being exposed in an individual with positive serological result (lower PLR).

Supplementary material Figure S2A,B show the variation of the misclassification error rate (MER) with the cut off used to determine seropositivity to PvMSP8. A misclassification error occurs when an exposed individual is classified by the serological tool as not exposed (false negative, FN), or a non-exposed individual is classified as exposed (false positive, FP). The cutoffs that minimize the MERs can also vary with the prevalence of exposure, showing high values (cut-off = mean plus 17 standard deviation of negative controls) with prevalence ~9% (survey prevalence) and lower values as prevalence increases (Supplementary material Figure S2). An increase in the importance (error cost) of FN relative to that of FP leads to a decrease in the cut-off that minimizes the misclassification cost rates (MCRs) (Supplementary material Figure S3).

## 3. Discussion

Here we show that specific human IgG response to *Plasmodium vivax* against the in-house baculovirus expressed recombinant protein PvMSP8, was a useful and effective SEM to identify recent *P. vivax* infections in the Peruvian Amazon. The high efficiency and yield of the in-house production of PvMSP8, its ability to trigger specific IgG antibody responses with good performance for detecting a recent *P. vivax* infection (Sn for infections ≤ 30 days >75%; overall Sp: 73.2%;), and the differences in the decay of anti-PvMSP8 IgG levels in comparison with the ones found for PvMSP10, suggest the potential use of IgG antibodies responses against PvMSP8 as a serosurveillance marker for recent *P. vivax* exposure in low-moderate transmission settings of the co-endemic Peruvian Amazon.

For this study, the production of PvMSP8 (54 KDa) was achieved through the baculovirus expression system with biological reactivity, presenting differential IgG antibodies responses to *P. vivax* infection between positive confirmed *P. vivax* individuals and healthy individuals, by ELISA. Thus, confirming the antigenicity of the PvMSP8 protein expressed in our laboratory. Choosing the right expression system for the production of a recombinant protein is an important phase since the intrinsic characteristics of the protein must be considered. The expression of recombinant erythrocyte invasion *Plasmodium spp.* proteins has been successfully reported in various systems, such as *Escherichia. coli* [17,18,19,20], cell-free system [21,22], mammalian cells [23,24], and baculovirus [25], among others [26,27,28,29,30]. Nevertheless, the existing data does not indicate that one heterologous expression system is definitely superior to others; and the identification of the optimal protein expression system remains an experimental test matter. The baculovirus expression system is currently a well-established methodology for the production of high molecular weight recombinant proteins. It was implemented in our laboratory [25], considering its relative ease and speed of escalation at which a biologically active protein can be expressed [31].

Previous studies have reported *Plasmodium* spp. protein production as MSP1 up to 0.85 mg/5.5 L and 0.9 mg/500 mL [32]. In this study, the production achieved for PvMSP8 was 0.428 mg/mL in a Sf9 cell culture volume of only 170 mL, demonstrating a high yield of in-house production and efficiency. However, to determine how much protein per liter of culture could be produced, bioprocessing scale-up will have to be achieved. Implementing the baculovirus expression system in our lab costed approximately $50,000 USD, including potential over-costs due to the need to import equipment and reagents not available in Peru. Although this cost seems high in comparison with commercial proteins ($3000 to $5000 USD cost for 500 µL of protein at 0.16 mg/mL, plus additional shipment and custom clearance fees), the investment is compensated by a constant protein production in only few weeks (once the process is standardized) and the avoidance of long times for the importation of products (three months or more).

IgG anti-PvMSP8 antibody responses were able to discriminate in a good manner between *P. vivax*–exposed and non-exposed individuals in the cohort study (AUC = 0.72); however, a slightly better performance was showed by PvMSP10 (AUC = 0.78). The evaluation of anti-PvMSP8 and anti-PvMSP10 IgG responses in combination did not allow for an improvement in performance indicators (AUC = 0.76 [95% CI = 0.67–0.86]) probing the redundancy between both antigens. It has been pointed out that not necessarily the proteins with the highest individual performances as SEM work best in combinations or that these combinations only show small improvements [8]. Moreover, during the population-based cross-sectional study, the performance of PvMSP8 as recent exposure marker of *P. vivax* infection in individuals with the antecedent of *P. vivax* infection in the past month and with a microscopy and/or qPCR positive *P. vivax* diagnosis at the time of plasma samples collection, showed an improvement and acceptable value (AUC = 0.79). Our results were similar to the ones reported recently by Longley et al., in 2020, where the use of a wheat-germ cell-free (WGCF)-expressed PvMSP8 recombinant protein was shown as part of a validated panel of SEM for detection of recent *P. vivax* infections, with a good performance (AUC = 0.69) on three longitudinal observational cohort studies, conducted in Thailand (AUC = 0.76), Brazil (AUC = 0.72) and Solomon Islands (AUC = 0.69) [8]. In this study the performance of PvMSP8 in conjunction with another seven SEM (RBP2b, RAMA, MSP1–19, Pv-fam-a, PvTRAg_28, EBPII and MSP3.10) reached 80% sensitivity and specificity [8]. We expect to improve the performance of PvMSP8 in combination with other proteins that already proved to be highly seroreactive in *P. vivax*-infected individuals from the Peruvian Amazon, such as PvMSP4, PvMSP7, Pv-fam-a, and PvAMA1, or the novel RPBP2 on future studies [14].

The intensity of malaria serological responses tend to increase with age as result of cumulative exposure to malaria. If this exposure is continuous and frequent, antibody levels can reach a plateau where no further increase is possible [8]. This could explain the better performance of serological measures in discriminating malaria exposure in areas of low transmission in comparison with the performance achieved in areas of moderate to high transmission (where antibody signals could be high and long lasting across all ages) [8,33].Therefore, we are not surprised by the finding that anti-PvMSP8 serological responses discriminated better in children than in adults in an area of moderate to high transmission like Mazan [24].

PvMSP8 was first described by Perez-Leal and collaborators in 2004 and since then only a few studies have evaluated its antigenicity [34]. Cheng et al., in 2017, produced a WGCF-PvMSP8 recombinant protein and demonstrated the high antigenicity of this protein (73.2% Sensitivity and 96.2% Specificity), in symptomatic, smear-positive individuals infected with *P. vivax* from Myanmar and Thailand using protein arrays [21]. In our cohort study, PvMSP8 as SEM identified individuals who had acquired *P. vivax* infections during the previous month with modest sensitivity (60%) and specificity (73%) using Luminex. A previous study that analyzed PvMSP10 antigenicity for the detection of *P. vivax*-infected individuals from Korea using serum samples and protein arrays, showed a sensitivity of 42% and specificity of 95% [35]. Interestingly, our results from the cohort study using the Luminex platform showed an increase in the sensitivity of PvMSP10 for detection of *P. vivax* exposure (sensitivity, 76.8% and specificity, 65.8%), in comparison with the results shown by Rosas-Aguirre et al., in 2020 (sensitivity, 58.1% and specificity, 81.8%), in which the same samples were processed by the ELISA method [15]. The Luminex high-throughput technology offers a wider dynamic range of detection than ELISA methodology [36,37,38] and; therefore, samples that might have been before classified as seronegative by ELISA methodology were classified as seropositive in our study. Nevertheless, the ELISA technique was chosen for the evaluation of PvMSP8 as SEM at the population level, in a first step to evaluate the potential implementation of this platform in health centers facilities in the Peruvian Amazon.

The differences between performances of SEM are not only due to the chosen protein expression systems or the method of antibody detection, but also to host genetic factors such as the magnitude of B cell signaling [39], antibody-secreting cells [40,41,42], antibody longevity [43,44], antibody subtypes [45], comorbidities [46,47], and nutritional status [48,49], which are beyond the scope of this study. It is likely that certain proteins will have more utility as serological markers in some endemic settings versus others [50] and according to the use-case scenario [7]. For example, it has been suggested that a use-case scenario of stratification of transmission will require a SEM with optimal balance between sensitivity and specificity for measurement of recent exposure across a range of transmission levels, which, in association with seroprevalence and georeferencing data, would be useful for the identification of hotspots and the implementation of targeting interventions on areas of highest transmission [7]. The evaluation of the impact of interventions from National Malaria Control Programs (NMCPs) would require specific SEM for measurement of recent exposure at the beginning of interventions and intermittently, in order to monitor the real effectiveness of the strategies [51,52]. Lastly, the documentation of absence of transmission will require SEM with high specificity for recent exposure and, depending on the elimination related goals, high species specificity [53].

Cheng et al., in 2017, showed that IgG antibody response to PvMSP8 was increased up to day seven post-infection, with a slight decrease after a month post-infection in Myanmar residents. Furthermore, IgG anti-PvMSP8 responses were found in plasma samples from 12-year recovered patients from China [21]. Kochayoo et al., in 2019, described that Thailand residents enrolled in a four-year cohort study showed long-term antibodies and Memory B Cells (MBCs) responses specific to PvMSP8 up to four years post-infection [54]. To our knowledge, there are no studies of antibody dynamics against PvMSP10. In this study, sensitivity of *P. vivax* exposure was highest for PvMSP8 (79%) and PvMSP10 (89%) on individuals with confirmed *P. vivax* infections occurring seven to 30 days before plasma sample collection; however, sensitivity decreased in relation to time since last documented infection. The sensitivity of PvMSP8 for detection of *P. vivax* exposure was highest on individuals with infections occurring seven to 30 days (72%) before plasma collection in comparison with individuals with infections occurring >60 days (53%) before plasma collection. Notably, high antibodies levels against PvMSP10 were maintained after 60 days since last documented infection in comparison with PvMSP8, pointing out a potential difference of antibody kinetics between these proteins that will need to be investigated in upcoming studies, which include follow-up of *P. vivax*-infected individuals over a longer time period and sampled at shorter time intervals.

We can visualize two specific scenarios where this protein can become a useful serological tool for the Malaria Zero Program [3]. The first one, the use of PvMSP8 alone and/or in combination with other SEMs in a highly sensitive point-of-contact antibody test for the detection of recent exposure events in a small sample size of targeted populations, such as high-risk groups who may harbor hypnozoites. The implementation of a decentralized immediate response would be based on the results of a robust and systematic use of this point-of-contact antibody test, which should be easy to use and easy to interpret [7]. A second scenario would be the application of serological testing and treatment interventions (seroTAT) based on the programmatic implementation of a highly sensitive and specific point-of-contact test, founded on PvMSP8 alone and/or in combination with other SEMs. Whereby individuals with history of recent exposure to *P. vivax* and a seropositive result would receive radical cure with liver-stage drugs (unless contraindicated or recent treatment can be confirmed). This alternative may be preferred over mass drug administration to avoid unnecessary treatments and the related risk of severe hemolysis in glucose-6-phosphate dehydrogenase-deficient individuals [55]. It is worth noticing that PvMSP8 is part of a recently validated protein panel for detection of recent exposure to *P. vivax*, proposed as a platform that can indirectly identify likely hypnozoite carriers that could be target for treatment of liver-stage drugs such as Primaquine or Tafenoquine. The results based on modeling the implementation of this seroTAT strategy would identify and treat at least 80% of likely hypnozoite carriers [8].

No seroreaction of PvMSP8 was found in plasma samples from Sengalese *P. falciparum*-infected individuals, pointing out the specificity of this protein for detection of *P. vivax* infections. Seroreaction against PvMSP8 was found in some plasma samples from *P. falciparum*-infected individuals from the Peruvian Amazon; nevertheless, significant differences were found between seroreaction results of these samples and *P. vivax*-infected individuals from the Peruvian Amazon, highlighting the probable presence of circulating antibodies resulting from previous exposure to *P. vivax* in this co-endemic region [15,25]. Unfortunately, one of the limitations of this study was the lack of information of history of previous *P. vivax* infections in these *P. falciparum* samples and the small sample number, due the low prevalence of *P. falciparum* infection in this region. Future studies will include the assessment of antibody longevity, antibody kinetics, and seroconversion in malaria infection, IgG subclasses, and MBC responses against PvMSP8, and its role, if any, in protection from infection. The possible role of IgM in the detection of acute infections in serosurveillance studies will also be evaluated. The design and study of smaller protein fragments or peptides of this protein, antigenic diversity, and performance upgrade in conjunction with other SEMs in terms of sensitivity, specificity, positive and negative likelihood ratio, misclassification error rate, and misclassification cost rates, according to different use case scenarios (prevalence) and quality assurance of protein production, should all be considered before its escalation and implementation as a point-of-contact antibody test in an ELISA or rapid diagnostic test format at local health facilities. Another limitation of the study was that we were unable to evaluate the performance of PvMSP8 as SEM in classifying symptomatic and asymptomatic individuals due to the low number of symptomatic cases. It would be interesting also to evaluate the functional role of antibodies against these proteins in *ex-vivo P. vivax* and *in-vitro P. falciparum* invasion studies.

## 4. Conclusions

The Baculovirus-produced PvMSP8 protein is a good recent exposure biomarker for the identification of populations affected by ongoing vivax malaria transmission in the Peruvian Amazon, even at low intensity, which is fundamental to malaria control and elimination efforts. The high efficiency and yield of our in-house baculovirus-produced PvMSP8 recombinant protein, and its good performance as SEM to *P. vivax* infection at a population level, make us think of a potential future scenario where the production of PvMSP8 can be scaled-up for its use as a SEM in a point-of-care (PoC) platform on a malaria control program level in the Peruvian Amazon.

## 5. Materials and Methods

### 5.1. Study Design

The present study was designed to evaluate IgG responses against an in-house Baculovirus-produced PvMSP8 recombinant protein as a biomarker of recent *P. vivax* exposure in the Peruvian Amazon. First, we measured total IgG responses against PvMSP8 and PvMSP10 proteins using a multiplex Luminex platform in a cohort of 422 Amazonian individuals with known history of *P. vivax* exposure, and the performance of both serological responses were compared in terms of their ability to discriminate between recently infected and not infected individuals. Then, the discriminatory ability of anti-PvMSP8 IgG responses (measured by ELISA) was evaluated at population level using 1251 plasma samples collected during a population-based cross-sectional survey in seven endemic riverine communities in the Peruvian Amazon.

### 5.2. Samples of Individuals with Known History of P. vivax Exposure (Cohort Study)

In January 2013, a population screening (PS) for blood sampling was carried out as a last point of collection of a 5-month longitudinal cohort study in two rural communities located in San Juan and Iquitos district, Cahuide (4.231° S, 73.487° W) and Lupuna (3.745° S, 73.323° W) (Figure 2). In the past two years, both communities had reported *P. vivax* and *P. falciparum* cases throughout the entire year (Pv/Pf ratio in 2012: 7.1/1), with a peak between February and July [56].

As previously described elsewhere [15], cohort participants (960 individuals) in these communities had a rigorous parasitological follow-up using microscopy between September 2012 and January 2013, by passive case detection (PCD), weekly active case detection of symptomatic individuals (wACDS), and monthly PS. Confirmed microscopic infections were immediately referred to health facilities for treatment following Peruvian national treatment guidelines [57]. Noteworthy, dried blood spots (DBSs) on filter paper were also collected during PS, and analyzed later by qPCR.

During the PS in January 2013, axillary temperature was taken from participants, and history of fever or any other malaria symptoms registered. Whole blood was collected by venipuncture in tubes with EDTA (BD Vacutainer, BD) and transported at 4 °C to the field lab where plasma and red blood cells separation by centrifugation (2205 g) took place; both components were stored at –70 °C until processing. Plasma samples were only thawed one time for inventory and a second time for ELISA processing.

A total of 422 individuals completed follow-up through January 2013 and had available plasma samples for serological analyses for this study, 258 individuals lived in Cahuide and 164 in Lupuna.

### 5.3. Samples Collected in a Population-Based Cross-Sectional Survey

A population-based cross-sectional survey was conducted in October 2018 in seven communities of the Mazan district: Gamitanacocha (3.428° S, 73.318° W), Libertad (3.496° S, 73.234° W), Primero de Enero (3.479° S, 73.199° W), Urco Miraño (3.361° S, 73.064° W), Yuracyacu (3.365° S, 72.989° W), Salvador (3.444° S, 73.155° W) and Puerto Alegre (3.510° S, 73.116° W) (Figure 2). Transmission in Mazan is unstable and seasonal, with a peak between May and September. *P. vivax* cases predominates over *P. falciparum* ones (Pv/Pf ratio: 3.5/1). A total of 1360 malaria cases were reported in 2018 [4,24].

After the measurement of axillary temperature and the registration of malaria-compatible symptoms from survey participants, finger-prick blood samples were taken for malaria screening by microscopy and to collect whole blood in microtainers with EDTA (Vacutest, Kima^®^, Padua, Italy). Confirmed microscopic infections were referred to health facilities for treatment [57]. The whole sample was centrifuged to separate red blood cells from plasma, and both components were transported at 4 °C to the field lab, to be stored at −20 °C until processing. Plasma samples were only thawed one time for inventory and a second time for ELISA processing. Red blood cells were analyzed by qPCR. A total 1251 individuals had available results by microscopy, qPCR, and ELISA.

### 5.4. Laboratory Procedures

#### 5.4.1. Microscopy

Thick and thin smears were stained for 10 min with a 10% Giemsa solution, and parasite density was computed after counting the number of asexual parasites for 200 white blood cells (WBCs) in the thick smear and assuming a concentration of 8000 WBCs/μL. Slides were read on-site and then again by a microscopy expert in our field laboratory at Iquitos city. A slide was declared negative if no malaria parasite was found after examining 100 fields [58]. Quality control was done blindly on all positive slides and 10% of randomly chosen negative slides by an expert microscopist from the Referential Laboratory of the Loreto Regional Health Direction (DIRESA). Discordant results were reassessed by a second senior expert microscopist.

#### 5.4.2. Real-Time Quantitative PCR (qPCR)

The QIAamp DNA Micro Kit from QIAGEN was used to extract DNA from ~6 mm diameter filter paper spots. The E.Z.N.A.^®^ Blood DNA Kit (Omega Bio-tek^®^, Norcross, GA, USA) was used to extract DNA from red blood cells following the manufacturer’s instructions with slight modifications (i.e., addition of TEN buffer (20 mM Tris-HCl, pH 8.0; 2 mM EDTA, pH 8.0; 0.2 M NaCl) supplemented with SDS 10% w/v). Extracted DNA was stored at 4 °C for immediate use and at −20 °C for later analyses.

Subsequent amplification was performed by a quantitative real-time PCR method targeting the 18s rRNA gene region. Following the protocol reported by Manrique et al. [59]. 5′-TAACGAACGAGATCTTAA-3′ and 5′-GTTCCTCTAAGAAGCTTT-3′ were used as primers, and qPCR conditions consisted of an initial denaturation at 95 °C for 2 min, followed by amplification for 45 cycles of 20 s at 95 °C, 20 s at 52 °C, and 30 s at 68 °C. Amplification was immediately followed by a melt program consisting of 5 s at 65 °C and a stepwise temperature increase of 0.5 °C/s up to 95 °C. The analysis of the differences in melting curves provided an accurate differentiation between Plasmodium species.

#### 5.4.3. Recombinant Proteins

PvMSP8 and PvMSP10 are immunogenic conserved proteins encoded by single copy genes (GenBank accession PVX_097625 and PVX_114145 respectively) expressed in the asexual blood stages of all *Plasmodium spp* species [60]. In this research, we tried to express PvMSP8 and PvMSP10 in our malaria laboratory at Universidad Peruana Cayetano Heredia, through an eukaryotic protein production system, Baculovirus, following procedures previously described by Bendezu et al. (Appendix A) [25]; however, the production of PvMSP8 was only successful. Of note, PvMSP8 expression did not include the signal and transmembrane domains. PvMSP10 was expressed in a human embryonic kidney (HEK293) mammalian cell line, as described elsewhere [15].

#### 5.4.4. Luminex

Antigens were coupled to carboxylate-modified microspheres (Luminex Corp., Austin, TX, USA) by covalent NHS-ester linkages via EDC (Biorad^®^, Hercules, CA, USA) and Sulfo-NHS (Biorad^®^) using the Bio-Plex^®^ COOH Beads Amine Coupling kit (Biorad^®^ Cat #171406001) following the manufacturer’s instructions. The total amount of antigens were 0.25 μg for PvMSP10 and 0.04 μg for PvMSP8 per 1.25 × 10^6^ beads. These antigen-coated microspheres were added to nonbinding 96-well plates (Bio-Plex Pro™ Flat bottom Plates) at 1000 beads per well (50 μL). Plasma samples were diluted 1:100 in PBT (PBS 1X, BSA 1% and Tween-20 0.05%) for measurement of total IgG. Next, 50 μL of diluted plasma samples were added and incubated with microspheres on a shaker for 30 min at room temperature in the dark. Microspheres were washed with PBT, and PE-conjugated anti-IgG (donkey F(ab’)2 anti-human IgG Fc R- PE, Jackson Immunoresearch Cat #709-116-098) at 1:100 dilution on PBT was added for incubation at 15 min in movement in the dark. The microspheres were washed and resuspended in 100 μL of buffer PBT and read on a Luminex^®^ 200™ (Luminex Corp.). Two standard curves of pooled plasma from seven infected individuals with mono-infection of *P. vivax* and seven infected individuals with mono-infection of *P. falciparum* (confirmed by qPCR), respectively, were included per plate as positive controls (dynamic range dilution of 1/50 to 1/102400) [61]. Pooled plasma from seven healthy individuals with no previous exposure to malaria (living in non-endemic areas, no reported trips to endemic areas and with confirmed negative qPCR malaria results) were used as negative controls. All control samples were diluted at 1:100 in PBT (PBS 1X, BSA 1% and Tween-20 0.05%). Data was screened to ensure adequate bead counts (>50) per antigen per sample, and median fluorescence intensity (MFI) values for sample/antigen pairs with adequate counts were only included in the analyses. The MFI mean value of sample pairs was corrected by subtracting from it the mean MFI values of technical blanks in each plate (wells without sample), and then log10 transformed (log_10_MFI).

#### 5.4.5. ELISA

Total IgG against PvMSP8 was detected on plasma samples using an ELISA protocol previously described [25] with slight modifications. Plasma samples were diluted in 1.5% non-fat milk washing solution (0.15 M Na2HPO4, 0.15 M NaH2PO4, 0.44 M NaCl, 0.05% of Tween20, and 0.05% of BSA) at 1:100. One hundred microliters of the diluted samples was added in duplicates to blocked ELISA plates coated separately with 0.5 μg/mL of PvMSP8 protein in Phosphate Buffered Saline (PBS) at pH 7.4. Plasma from two confirmed infected individuals (either by *P. vivax* or *P. falciparum*) were used as positive controls. Pooled plasma from five healthy individuals with no previous exposure to malaria (living in non-endemic areas, no reported trips to endemic areas and with confirmed negative qPCR malaria results) were used as negative controls. All control samples were diluted at 1:100 in 1.5% non-fat milk washing solution and included in the plate by duplicated. Peroxidase AffiniPure Goat Anti-Human IgG, Fcγ fragment specific (Jackson Immuno Research, West Grove, PA, USA; Cat # 109-035-098), diluted 1:2000, was used as a secondary antibody (100 μL/well) and incubated for 1 h before development of the ELISA using 100 μL TMB peroxidase substrate (BD OptEIA). The reaction was stopped using 50 μL/well of 0.25 M HCl. The optical density (OD) values in antigen-coated wells were read at 450 nm (ELISA iMARK microplate-reader, BioRad) and corrected by subtracting from them the mean of OD values in blank wells (were plasma samples was replaced with 1.5% non-fat milk washing solution).

PvMSP8 antigenicity was preliminarily assessed using 40 samples of individuals with confirmed *P. vivax* mono-infection by qPCR, and compared to 40 samples of healthy individuals with no history of malaria infection and negative qPCR to malaria. Another assay was performed to assess potential cross-reactivity between recombinant PvMSP8 and *P. falciparum* plasma. To this end, antigen-antibody responses obtained from 20 plasma samples of Senegalese adult subjects with documented *P. falciparum* infection in the past 2–3 months were compared to those obtained from 20 samples of microscopically confirmed *P. falciparum*-infected individuals from the Peruvian Amazon, 20 samples of qPCR-confirmed *P. vivax*-infected individuals from the Peruvian Amazon, and 20 samples from healthy individuals.

#### 5.4.6. Data Management and Statistical Analysis

Demographic, parasitological and serological data were double-entered and crosschecked in Excel (Microsoft Corp., Seattle, WA, USA). Analyses were done using R v.2.15 software (R Development Core Team, R Foundation for Statistical Computing, Vienna, Austria) and GraphPad Prism v. 8.0 (GraphPad Software Inc., San Diego, CA, USA).

For the evaluation with samples from the cohort study, *P. vivax* malaria exposure was defined as an individual with the confirmed presence of *P. vivax* parasites in the blood by microscopy and/or qPCR at any time during the 5-month cohort follow-up. When more than one *P. vivax* infection was detected in an individual during the follow-up, only the last detected infection was considered in the analysis. For the evaluation with samples from the cross-sectional survey, *P. vivax* exposure was defined as an individual with positive *P vivax* parasitological result by microscopy and/or qPCR at the time of the survey. This definition was later extended to also consider individuals who had a microscopically- confirmed *P. vivax* malaria episode in the past month (antecedent of malaria).

Cross-validation logistic models assessed the relationship between antigen-antibody responses (i.e., log_10_MFI or ODs) and *P. vivax* exposure status (exposed/non-exposed) using a resampling method (100 times), whereby two-thirds of plasma samples were randomly selected to be in a training data set, and the remaining one-third in the testing data set. Cross-validated receiver operating characteristic (ROC) curves were averaged using the R package ROCR to characterize the sensitivity/specificity tradeoffs for the binary classifier. Areas under the curve (AUCs) with corresponding DeLong 95% CI [62] assessed the model discriminatory efficiency.

For each sample, the difference between the log_10_MFI of each sample and the log_10_MFI mean of negative controls (in case of Luminex assays), or between the OD of each sample and the OD mean of negative controls (in case of ELISA assays), were calculated. These differences were expressed as a number of standard deviations (SDs) from the corresponding mean value of negative controls, allowing for the dichotomized classification of samples as either seropositive (greater than or equal to cutoff of 3 SDs) and seronegative (less than cutoff of 3 SDs) [15]. The Mann–Whitney test was used to evaluate the differences in the OD values among the groups for the evaluation of antigenicity and cross-reaction of PvMSP8. Data with a *p* value < 0.05 were considered statistically significant.

Box plots and Kruskal–Wallis test with Dunn’s multiple comparison post-hoc compared quantitative antibody responses between the exposed group (the whole group or sub-divided groups according to the time since last *P. vivax* infection) and the non-exposed group. Moreover, performance indicators of dichotomized serological results (seropositive or seronegative) for the identification of malaria exposure (i.e., sensitivity (Sn), specificity (Sp), positive predictive value (PPV), and negative predictive value (NPV)) were calculated, and their two-sided 95% confidence intervals (CIs) estimated using the Wilson score method.

Further assessments included the simulation of different cutoffs for PvMSP8 seropositivity (using ELISA serological data) and the determination of the ones (number SDs from the mean value of negative controls) that minimize misclassification error rates (MERs) in comparison with assumed gold standard of *P. vivax* exposure. MERs can be calculated as the sum of false negatives (FNs) and false positives (FPs) divided by the total number of tested individuals, assuming no difference in importance (error costs) between both diagnosis errors (FN = FP). An alternative formula for MERs makes more evident the relationship of this indicator with the prevalence of exposure: *pFN* × *prev* + *pFP* × (1 − *prev*), where *pFN* is the proportion of FN among individuals with the event, *pFP* is the proportion of FP among individuals without the event, and *prev* is the prevalence of the event. When FN and FP are assumed to have different error costs (FN ≠ FP ratio; i.e., a higher importance of one error type over the other one), misclassification cost rates (MCRs) were calculated similar as for MERs, considering the differential error costs for FNs and FPs. In addition, plots showed the variation of Sn, Sp, positive likelihood ratio (PLR), and negative likelihood ratio (NLR) with the cut-off used to determine seropositivity.

## Figures and Tables

**Figure A1:**
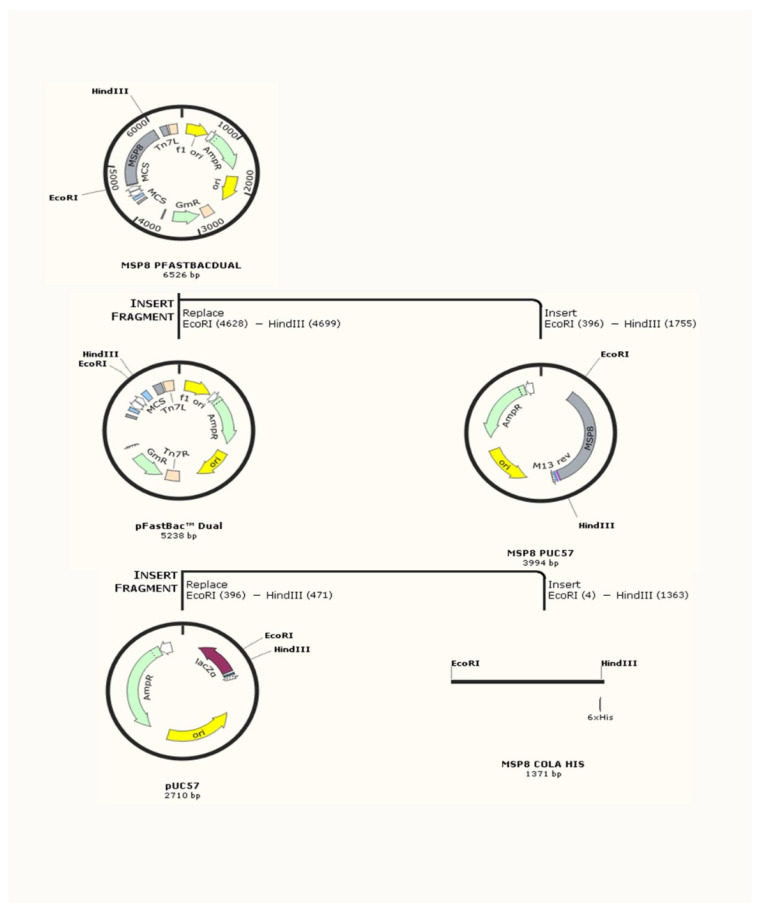
Design strategy of Baculovirus expression in Sf9 insect cells of recombinant protein PvMSP8. PvMSP8 gene with a His tag was cloned in to a pUC57 vector and then sub-cloned in to pFastBacDual vector using EcoRI and HindIII.

## Supplementary Materials

The following are available online at https://www.mdpi.com/2076-0817/10/3/282/s1, Table S1: Baseline characteristics of cohort participants. Table S2: Baseline characteristics of cross-sectional study participants. Table S3: Participants from the cross-sectional study classified by age groups and exposure condition. Figure S1: Variation of sensitivity (Sn), specificity (Sp), positive likelihood ratio (PLR), and negative likelihood ratio (NLR), with the cutoff used to determine seropositivity. Figure S2: Misclassification error rates (MERs) of dichotomized serological responses to PvMSP8, in discriminating *P. vivax* exposure. Figure S3: Misclassification error rates (MERs) and misclassification cost rates of dichotomized serological responses to MSP8 in discriminating *P. vivax* exposure

## Appendix A

**Design and production of recombinant *Plasmodium vivax* MSP8 Protein**

Design

MODB (Plasmodb.org) was the source for the amino sequences for recombinant protein design. The sequence of *P. vivax* MSP8 was based on strain Sal I.

The post-signal amino acid sequences of PvMSP8 (PVX_097625) were synthesized in Sf9 insect cell-optimized codons and cloned into the pFastBacDual expression plasmid. Signal peptides were not included in the recombinant protein, which was engineered to have a His-Tag for purification.
PLAS PVX_097625 PvMSP8 amino acid sequence (highlighted in yellow), including His-tag (underlined)
MEGNVSPPNFNDNRVNGNNGNKGNGNDNDVPSFIGGNNNNVNGNNDDNIFNKNGKDVTRNDGDAKDGENRNNKKNENGSGSNENNSIANADNGSGKSDANANQIDEDGNKMDEASLKKILKIVDEMENIQGLLDGDYSILDKYSVKLVDEDDGETNKRKIIGEYDLKMLKNILLFREKISRVCENKYNKNLPVLLKKCSNVDDPKLSKSREKIKKGLAKNNMSIEDFVVGLLEDLFEKINEHFIKDDSFDLSDYLADFELINYIIMHETSELIDELLNIIESMNFRLESGSLEKMVKSAESGMNLNCKMKEDIIHLLKKSSAKFFKIEIDRKTKMIYPVQATHKGANMKQLALSFLQKNNVCEHKKCPLNSNCYVINGEEVCRCLPGFSDVKIDNVMNCVRDDTLDCSNNNGGCDVNATCTLIDKKIVCECKDNFEGDGIYCGHHHHHH
PVX_097625 PvMSP8 DNA sequence (highlighted in yellow), including His-tag (underlined) and EcoRI (in red) and HindIII (in green) restriction sites for cloning in to pFastBac expression vector.
GAATTCGGCATGGAAGGAAACGTTAGCCCACCCAACTTTAATGACAACAGGGTAAACGGCAACAATGGAAATAAAGGCAACGGAAATGACAACGACGTGCCGTCGTTCATTGGAGGAAACAATAATAACGTGAACGGCAATAATGATGATAACATTTTTAATAAAAATGGAAAGGATGTCACCCGAAATGATGGCGATGCAAAGGATGGAGAAAATCGAAATAACAAGAAAAACGAAAATGGCAGTGGCTCCAATGAGAATAACTCCATTGCAAATGCGGACAATGGTAGCGGCAAATCTGATGCGAATGCCAACCAAATTGATGAGGATGGAAATAAAATGGATGAAGCATCTTTAAAGAAAATCCTCAAAATTGTAGACGAAATGGAAAATATTCAAGGACTGCTCGATGGAGATTACAGCATTTTGGATAAGTACAGTGTCAAATTAGTTGATGAAGATGATGGAGAAACGAATAAAAGAAAAATCATTGGAGAATATGATTTGAAAATGTTAAAAAATATTTTATTGTTCAGAGAAAAAATTTCCCGAGTTTGTGAAAATAAATACAATAAAAATTTACCCGTCTTGTTAAAAAAATGCTCAAATGTGGATGACCCCAAATTGAGTAAATCCAGGGAAAAAATTAAAAAAGGATTAGCAAAAAATAATATGAGCATTGAAGATTTTGTGGTAGGTTTGTTGGAAGATTTATTTGAGAAAATTAATGAACATTTTATTAAAGACGATTCATTTGATTTGAGTGACTATTTAGCCGATTTCGAGCTCATCAATTATATAATTATGCACGAAACGTCCGAATTGATCGATGAGCTTTTGAACATAATAGAGTCCATGAATTTCAGATTGGAATCCGGATCTTTGGAGAAAATGGTTAAATCTGCAGAATCAGGAATGAACTTAAATTGCAAAATGAAGGAAGACATAATTCACTTACTTAAGAAATCCTCCGCCAAATTTTTTAAAATCGAAATTGACAGAAAGACCAAGATGATATACCCAGTGCAGGCTACACACAAAGGTGCCAACATGAAACAACTCGCCCTGAGCTTCCTCCAGAAGAACAATGTATGTGAACATAAAAAGTGCCCATTGAACTCCAACTGCTATGTTATAAATGGAGAGGAGGTCTGCAGATGTCTACCCGGATTTAGCGACGTCAAAATTGATAACGTGATGAACTGCGTTAGGGATGATACCCTTGACTGTAGCAACAACAACGGTGGCTGTGATGTGAACGCAACGTGTACCCTTATAGACAAAAAAATTGTGTGTGAATGCAAGGACAACTTTGAGGGAGACGGAATATACTGCGGCCATCATCACCATCACCACTAGAAGCTT
