# Supplementary material for: PvMSP8 as a Novel Plasmodium vivax Malaria Sero-Marker for the Peruvian Amazon
Source: Pathogens. 2021 Mar 2;10(3):282. doi: 10.3390/pathogens10030282 (PMC7999794; doi:10.3390/pathogens10030282)

Supplemental Material

Table S1. Baseline characteristics of cohort participants (N = 422)						
	**Total**		**Cahuide**		**Lupuna**	
	N	%	n=258	%	n=164	%
**Gender**						
Female	232	55.1	143	55.4	90.0	54.9
Male	189	44.9	115	44.6	74	45.1
**Age groups (years)**						
<15	219	51.9	129	50.0	90	54.9
>15	203	48.1	129	50.0	74	45.1
**Main occupation (≥18 years)**						
None	82	19.4	45	17.4	37	22.6
Labourer	23	5.5	18	7.0	5	3.0
Logger or lumberjack	14	3.3	11	4.3	3	1.8
Fishermen	0	0.0	0	0.0	0	0.0
Breeder	4	0.9	4	1.6	0	0.0
Farmer	49	11.6	31	12.0	18	11.0
Trader	15	3.6	11	4.3	4	2.4
Housewife	65	15.4	39	15.1	26	15.9
Student	168	39.8	98	38.0	70	42.7
Other	2	0.5	1	0.4	1	0.6

Table S2. Baseline characteristics of cross-sectional study participants (N=1,251)

			Primero de Enero		Urco Mirano		Libertad		Puerto Alegre		Salvador		Gamitanacocha		Lago Yuracyacu	
	**Total**	**%**	n=93	%	n=223	%	n=230	%	n=192	%	n=334	%	n=70	%	n=109	%
**Gender**																
**Female**	643	51.4	41	44.1	109	48.9	112	48.7	98	51.0	182	54.5	44	62.9	57	52.3
**Male**	608	48.6	52	55.9	114	51.1	118	51.3	94	49.0	152	45.5	26	37.1	52	47.7
**Age groups (years)**																
**<15**	634	50.7	45	48.4	117	52.5	101	43.9	103	53.6	178	53.3	33	47.1	57	52.3
**≥15**	617	49.3	48	51.6	106	47.5	129	56.1	89	46.4	156	46.7	37	52.9	52	47.7
**Main occupation (≥18 years)**																
**None**	257	20.5	15	16.1	43	19.3	65	28.3	29	15.1	61	18.3	19	27.1	25	22.9
**Labourer**	1	0.1	0	0.0	0	0.0	0	0.0	0	0.0	0	0.0	0	0.0	1	0.9
**Logger or lumberjack**	6	0.5	1	1.1	0	0.0	2	0.9	3	1.6	0	0.0	0	0.0	0	0.0
**Fishermen**	16	1.3	0	0.0	0	0.0	3	1.3	3	1.6	0	0.0	0	0.0	10	9.2
**Breeder**	1	0.1	0	0.0	0	0.0	0	0.0	0	0.0	0	0.0	0	0.0	1	0.9
**Farmer**	435	34.8	35	37.6	76	34.1	95	41.3	68	35.4	111	33.2	29	41.4	21	19.3
**Trader**	5	0.4	0	0.0	0	0.0	0	0.0	1	0.5	2	0.6	0	0.0	2	1.8
**Housewife**	53	4.2	3	3.2	4	1.8	14	6.1	8	4.2	15	4.5	2	2.9	7	6.4
**Student**	465	37.2	38	40.9	97	43.5	49	21.3	79	41.1	141	42.2	20	28.6	41	37.6
**Other**	12	1.0	1	1.1	3	1.3	2	0.9	1	0.5	4	1.2	0	0.0	1	0.9

Table S3. Participants from the cross-sectional study classified by age groups and exposure condition (N=1,251)

				**Primero de Enero**		**Urco Mirano**		**Libertad**		**Puerto Alegre**		**Salvador**		**Gamitanacocha**		**Lago Yuracyacu**	
		**Total**	**%**	n=93	%	n=223	%	n=230	%	n=192	%	n=334	%	n=70	%	n=109	%
**Age groups (years)**	**First definition***																
**<15**	Non-exposed	603	48.2	42	45.2	115	51.6	85	37.0	100	52.1	176	52.7	28	40.0	57	52.3
	Exposed	31	2.5	3	3.2	2	0.9	16	7.0	3	1.6	2	0.6	5	7.1	0	0.0
**≥15**	Non-exposed	567	45.3	44	47.3	103	46.2	109	47.4	83	43.2	1 51	45.2	28	40.0	49	45.0
	Exposed	50	4.0	4	4.3	3	1.3	20	8.7	6	3.1	5	1.5	9	12.9	3	2.8
	**Second definitionº**																
**<15**	Non-exposed	587	46.9	40	43.0	115	51.6	76	33.0	100	52.1	176	52.7	24	34.3	56	51.4
	Exposed	47	3.8	5	5.4	2	0.9	25	10.9	3	1.6	2	0.6	9	12.9	1	0.9
**≥15**	Non-exposed	553	44.2	40	43.0	103	46.2	104	45.2	80	41.7	151	45.2	27	38.6	48	44.0
	Exposed	64	5.1	8	8.6	3	1.3	25	10.9	9	4.7	5	1.5	10	14.3	4	3.7
	**Malaria antecedent (past month)**																
**<15**	No	616	49.2	43	46.2	117	52.5	90	39.1	103	53.6	178	53.3	29	41.4	56	51.4
	Yes	18	1.4	2	2.2	0	0.0	11	4.8	0	0.0	0	0.0	4	5.7	1	0.9
**≥15**	No	603	48.2	44	47.3	106	47.5	124	53.9	86	44.8	156	46.7	36	51.4	51	46.8
	Yes	14	1.1	4	4.3	0	0.0	5	2.2	3	1.6	0	0.0	1	1.4	1	0.9

* First definition of *P. vivax* exposure was a positive *P. vivax* result (by microscopy and/or PCR) at the time of sample collection

° Second definition included individuals with a positive result and/or the antecedent of *P. vivax* malaria in the past month (red line).

Figure S1: Variation of sensitivity (Sn), specificity (Sp), positive likelihood ratio (PLR) and negative likelihood ratio (NLR) with the cutoff used to determine seropositivity.

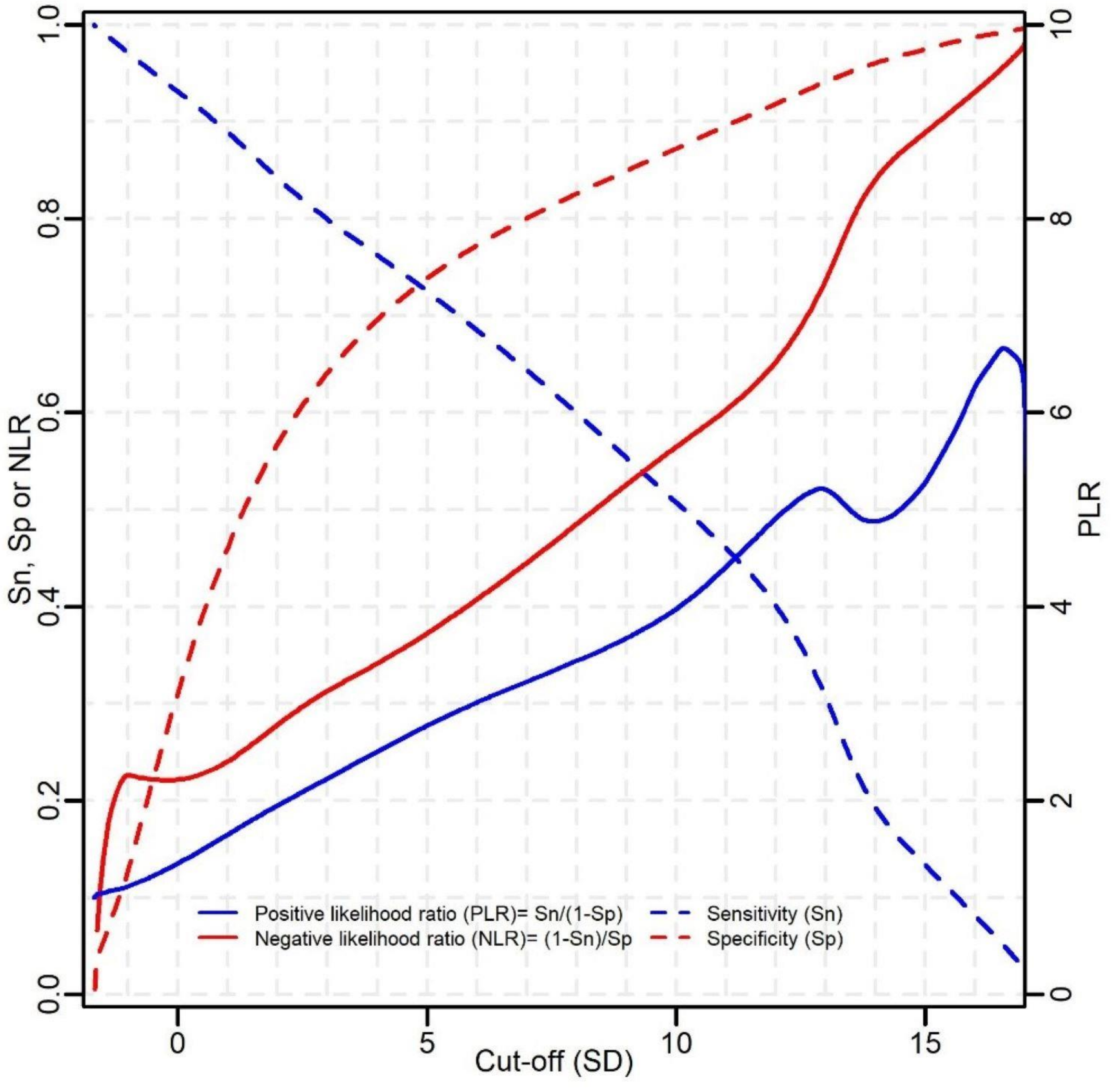

Figure S2: Misclassification/error rates (MER) of dichotomized serological responses to PvMSP8, in discriminating *P. vivax* exposure. (A) Variation of MER with cutoff used to determine seropositivity; (B) variation of false negatives (FN) and false positive (FP) rates with the cutoff used to determine seropositivity.

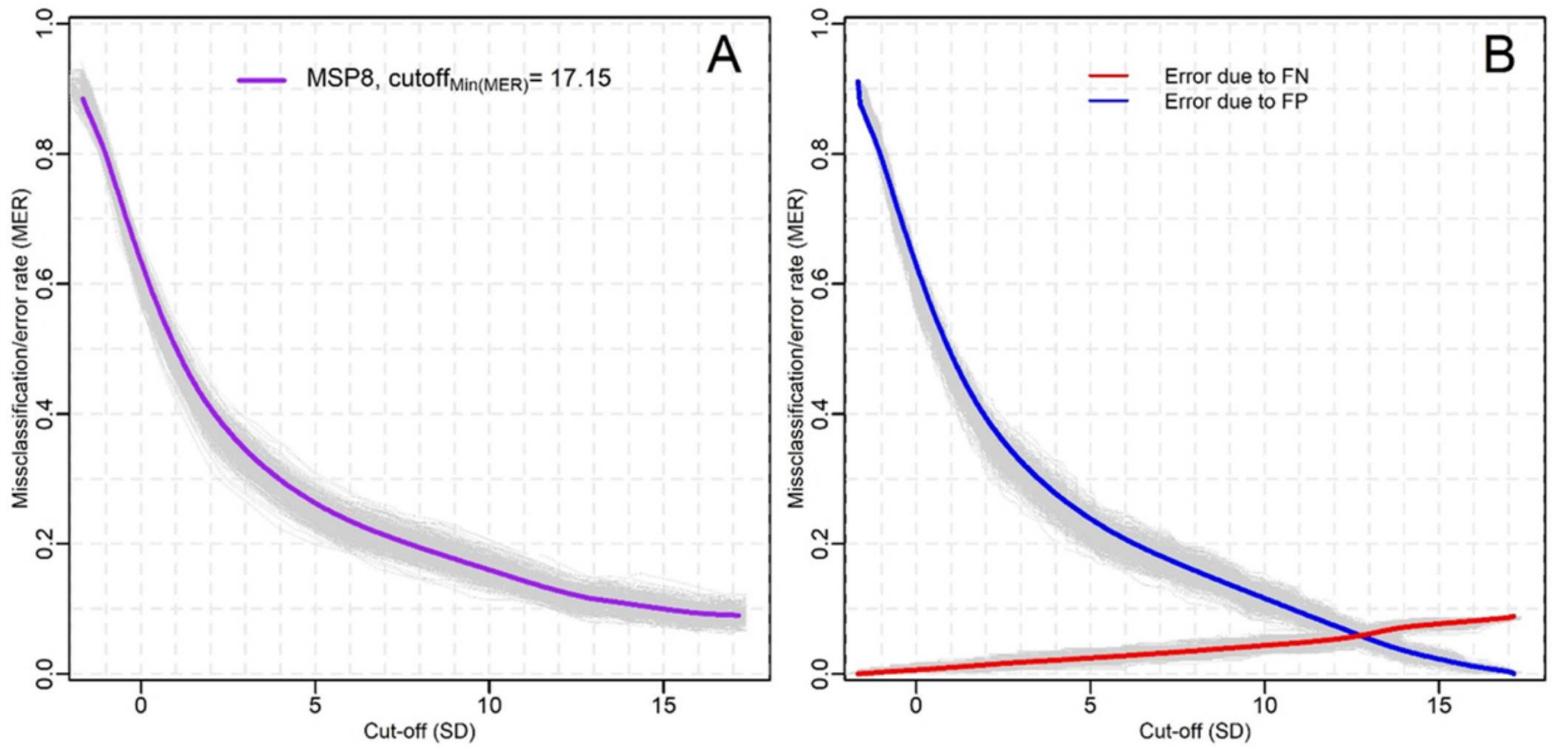

Figure S3: Misclassification/error rates (MER) and misclassification cost rates of dichotomized serological responses to MSP8 in discriminating *P. vivax* exposure: **(**A) Variation of MER with the cutoff used to determine seropositivity, assuming different *P. vivax* exposure prevalence; (B) variation of MCR with cutoff used to determine seropositivity, assuming different cost ratios between false negatives (FN) and false positives (FP).

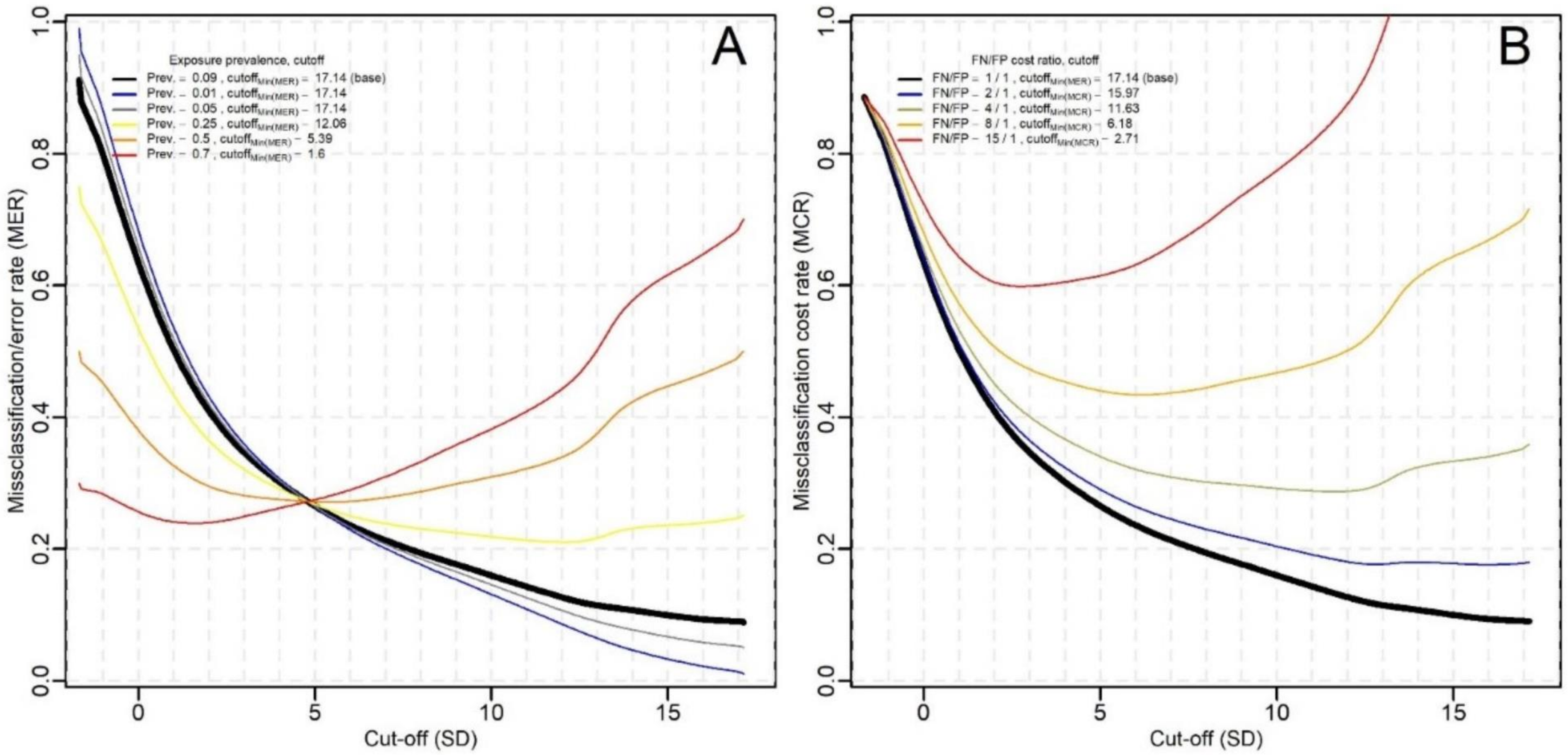

Supplement: Supplementary file 1 [file pathogens-10-00282-s001.pdf]
